# Tracking the Origin and Deciphering the Phylogenetic Relationship of Porcine Epidemic Diarrhea Virus in Ecuador

**DOI:** 10.1155/2017/2978718

**Published:** 2017-12-12

**Authors:** Maritza Barrera, Ana Garrido-Haro, María S. Vaca, Danilo Granda, Alfredo Acosta-Batallas, Lester J. Pérez

**Affiliations:** ^1^Facultad de Ciencias Veterinarias, Universidad Tëcnica de Manabí, Ave Urbina y Che Guevara, Portoviejo, Manabí, Ecuador; ^2^Laboratorio de Biología Molecular, Agencia Ecuatoriana de Aseguramiento de Calidad del Agro (Agrocalidad), Av. Interoceánica, Km. 14.5, La Granja MAGAP, Tumbaco, Pichincha, Ecuador; ^3^Laboratorio de Epidemiologia y Bioestadistica Veterinária, Universidad de São Paulo, São Paulo, SP, Brazil; ^4^Dalhousie Medicine New Brunswick (DMNB), Saint John, NB, Canada E2L 4L5

## Abstract

In 2010, new Chinese strains of porcine epidemic diarrhea virus (PEDV), clinically more severe than the classical strains, emerged. These strains were spread to United States in 2013 through an intercontinental transmission from China with further spreading across the world, evidencing the emergent nature of these strains. In the present study, an analysis of PEDV field sequences from Ecuador was conducted by comparing all the PEDV S gene sequences available in the GenBank database. Phylogenetic comparisons and Bayesian phylogeographic inference based on complete S gene sequences were also conducted to track the origin and putative route of PEDV. The sequence from the PED-outbreak in Ecuador was grouped into the clade II of PEDV genogroup 2a together with other sequences of isolates from Mexico, Canada, and United States. The phylogeographic study revealed the emergence of the Chinese PEDV strains, followed by spreading to US in 2013, from US to Korea, and later the introduction of PEDV to Canada, Mexico, and Ecuador directly from the US. The sources of imports of live swine in Ecuador in 2014 were mainly from Chile and US. Thus, this movement of pigs is suggested as the main way for introducing PEDV to Ecuador.

## 1. Introduction

Porcine epidemic diarrhea (PED) is an acute and highly contagious disease, which causes severe enteritis, vomiting, watery diarrhea, dehydration, and high mortality rates in pigs [1]. PED was first described in 1971 in United Kingdom, affecting fattening pigs [2], and the etiological agent was identified in Belgium as a new coronavirus, which was designated as PED virus (PEDV) [3]. PEDV belongs to the genus* Alphacoronavirus* of the family Coronaviridae, subfamily Coronavirinae, and order Nidovirales. PEDV genome consists of 28 kb long single-stranded RNA with positive polarity and includes seven known open reading frames (ORFs). Two large ORFs, 1a and 1b, occupying two-thirds of the genome, encode two nonstructural polyproteins (pp1a and pp1b) that direct genome replication and transcription. The remaining one-third of the genome encodes four structural proteins, spike (S), envelope (E), membrane (M), and nucleocapsid (N), and one hypothetical accessory protein encoded by the ORF3 gene [4]. Of all viral proteins, the PEDV S protein has a pivotal function regulating interactions with specific host-cell receptor glycoproteins to mediate viral entry [5]. Therefore, PEDV S protein has been often used to understand the genetic relationships between different PEDV strains and the epidemiological status of PEDV in the field (reviewed in [4]). Other genes including ORF3, E, M, or N have been also utilized for phylogenetic inference [6–9] and some studies have included PEDV full genome sequences to get better phylogenetic resolution [10, 11]. However, realistically, sequencing the full genome of PEDV is still expensive, from both computational and laboratory perspectives. Moreover, for many computationally intensive analyses, utilizing the full genome is unfeasible. It would be, therefore, beneficial to use only those genomic regions that contain the highest phylogenetic signal to reduce cost without losing valuable information [12]. In fact, phylogenetic markers together with powerful Bayesian phylogenetic approaches have been successfully applied to track the origin of important viral outbreaks [13, 14] and establish molecular epidemiology links among different viral strains including coronavirus members [15]. For PEDV it was recently shown that the S and nsp3 genes contain the lowest phylogenetic noise; therefore both are the highest recommended for phylogenetic analysis and molecular characterization studies [11].

In 2010, new Chinese strains of PEDV clinically more severe than the classical strains emerged [16]. These strains were spread to United States in 2013 through an intercontinental transmission from China [17] with further spreading to Canada [18], Mexico, and other countries into the American continent including Colombia and Dominican Republic (reviewed in [1]). Moreover, in Europe, the recent reports in Germany [19], France [20], and Belgium [21] of PED-outbreaks caused by strains closely related to the variants identified in China and the United States evidence the emergent nature of these strains. The epidemiological situation regarding PEDV turned more complicated since in December 2013 a second PEDV strain OH851 (later named as S-INDEL strain) emerged in the US [22]. This new PEDV strain was also reported in spring 2014 in Germany [19, 23], as a consequence of a probable single or simultaneous introduction [24] and with later reports in other European countries including France [20], Belgium [21], Italy [25], Austria [26], and Spain [27]. Even though the PEDV S-INDEL strains have shown lower virulence in the field [28] the clinical manifestations of the disease in the European countries affected have been very variable, with ranges of mortality between 0 and 70% (reviewed in [29]). This recent global reemergence of PED requires urgent attention and deeper understanding of PEDV epidemiological links driving the changes in viral pathogenicity. Therefore, this report was conducted using the complete sequence of the S gene and powerful Bayesian phylogeographic reconstructions to clarify the putative origin of PEDV in Ecuador, revealing the wide expansion of the emergent PEDV strains, which caused the first PEDV outbreak in this country.

## 2. Material and Methods

### 2.1. Ethics Statement

International standards for animal welfare were used for all animal samples collected, following the regulations for animal sampling of the article number 134 of Animal Welfare Law included into the National Constitution of the Republic of Ecuador.

### 2.2. Collection, Selection, and Processing of Samples

On July 2014, a clinical outbreak with epidemiological characteristics compatible with PEDV was reported in a pig farm located in the province of Cotopaxi, Ecuador. In detail, the commercial farm contained a total of 10,909 animals from which 1,341 animals were affected, showing clinical signs compatible with PED, 1,043 died as a consequence of the disease, and 1,401 were slaughtered as control measure. To avoid further spreading of the disease a National Contingency Plan was applied including restrictions on the animal movement, increasing the biosafety measures, and establishment of epidemiological surveillance at national level. From the PED-outbreak a viral isolation was obtained and named PEDV/Cotopaxi/2014. A total of five fragments of ileum from three different diarrheic pigs were taken together with the viral isolate PEDV/Cotopaxi/2014 for total RNA isolation from mucosal scrapings and cell supernatant, respectively. RNA isolation was performed using TRIzol reagent (Invitrogen) following the manufacturer's directions with modifications to ensure a high RNA yield and quality.

### 2.3. Amplification and Sequencing of Complete S Gene of PEDV

A total of thirteen primers were designed using the Primer 3Plus software [32] to amplify five overlapping fragments of the S gene (Table 1) covering the complete S gene: 218 bases before the start codon and 213 bases after the termination codon of strain TC PC170-P2 virus PED United States (accession number GenBank: KM392227). All segments were amplified using SuperScript® III One-Step RT-PCR High Fidelity System with Platinum® Taq DNA Polymerase (Invitrogen) following the manufacturer's directions. The amplicons were visualized in agarose gel and purified by Pure Link Kit Quick Gel Purification (Invitrogen) following the manufacturer's instructions. The resulting products were submitted to bidirectional DNA sequencing using BigDye Terminator cycling conditions by an external laboratory (Macrogen, Korea). Consensus sequences were generated using the software Sequencher version 6.1, 2014 (Code Gene Corporation). Nucleotide BLAST analysis (https://www.ncbi.nlm.nih.gov/blast/Blast.cgi) was initially used to verify the identity of the sequences obtained.

### 2.4. Sequence and Phylogenetic Analyses

To perform sequence comparison analyses and to establish the phylogenetic relationships of PEDV sequence from Ecuador, alignments using the consensus sequence of complete S gene available at GenBank database (Supplementary Information Table S1) were conducted by the algorithm ClustalW included in the program BioEdit Sequence Alignment Editor [33]. To remove sequences with a possible recombinant event from the alignment datasets, searches for recombinant sequences and crossover regions were performed using Geneconv, RDP, MaxChi, Chimera, BootScan, SiScan, 3Seq and LARD, all implemented in RDP3 Beta 4.69, as previously described in Alfonso-Morales et al. [12]. The software jModelTest 2.0 [34] was used to estimate the best-fit model using the Akaike (AIC) and Bayesian information criteria (BIC). The best-fit models for the complete S gene were selected. Phylogenetic analyses were performed by Bayesian Inference (BI), Neighbour-Joining (NJ), and Maximum Likelihood (ML) methodologies as described elsewhere in [35]. All topologies obtained were compared as described by Alfonso-Morales et al. [12]. To visualize the structure of S protein and denote amino acids changes, a model of the S protein was kindly provided by Professor Douglas Marthaler from Department of Veterinary Population Medicine, College of Veterinary Medicine, University of Minnesota, St. Paul, MN, USA, and recreated using Chimera software package (http://www.cgl.ucsf.edu/chimera).

### 2.5. Phylogeographic Analysis

A discrete phylogeographic analysis (DPA) was conducted using a standard continuous-time Markov chain (CTMC) model with Bayesian stochastic search variable selection (BSSVS) to model the geographic transmission of PEDV from the selected sequence of the dataset (Supplementary Material Table S1, sequences denoted by asterisk) as described by Alfonso-Morales et al. [14]. Briefly, the Bayesian Markov chain Monte Carlo (MCMC) analysis was performed in two independent runs. The resulting maximum clade credibility (MCC) phylogenetic tree was obtained by TreeAnnotator and summarized using the SPREAD software [36]. A Keyhole Markup Language (KML) file was generated to identify the major routes of geographic diffusion. The Bayes factor (BF) test was used to select the most probable routes of transmission. The resulting KML files from SPREAD with a nonzero expectancy that showed a BF > 5 were visualized by Google Earth (available at: https://earth.google.com/).

## 3. Results and Discussion

After the PEDV outbreak in the United States in 2013, followed by the fast spreading of the virus to Canada, Mexico, Korea, and Taiwan, an important turn in PED research has taken place [1]. Thus, a relevant increase of studies about the epidemiology, genetic structure, and characteristics of PEDV has occurred to get a better understanding of this disease, which is currently the most fatal in pigs and one of the economic concerns for the pig industry [4].

**Figure 1:**
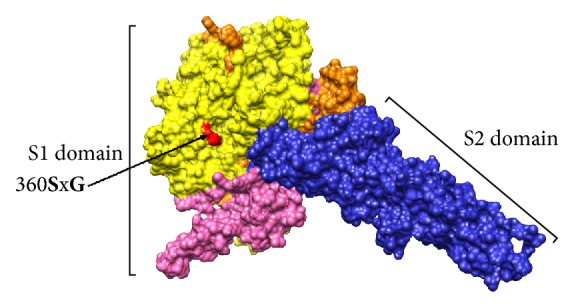
*3D-Representation of the monomer model of the PEDV S protein.* The model was kindly provided by Dr. Marthaler published in Jarvis et al. [30]; Chimera software v1.6.2 was used for visualization. Domains S1 (orange) and S2 (blue) are denoted. The C-terminal RBD of the S1-domain is represented in pink; N-terminal RBD of S1 domain is highlighted in yellow. The mutation 360SxG found in PEDV/Cotopaxi/2014 is denoted and represented in red.

In the present study, an analysis of PEDV field sequences from Ecuador was conducted by comparison with all the PEDV S gene sequences available in the GenBank database. In addition, phylogenetic comparisons and Bayesian phylogeographic inference based on complete S gene sequences were conducted to track the origin and putative route of PEDV. The S gene of PEDV has more than 4000 nucleotides and encodes the S protein, which constitutes the spikes of the viral envelope, responsible for its high variability. The PEDV S glycoprotein is known to be an appropriate viral gene for determining the genetic relatedness among PEDV isolates [4]. The sequences obtained from the animals selected and the viral isolate PEDV/Cotopaxi/2014 were assembled, yielding a final fragment of 4414 nt of length for each one, all of them containing the 4160 nt of the complete S gene. The identity of the sequences obtained was 100% between them; therefore, to avoid redundant entries at GenBank database, the sequences were submitted as a single entry under the accession number KT336490. The genetic identity of the sequence KT336490 (thereafter mentioned as PEDV/Cotopaxi/2014) was initially inferred from BLASTn analysis sharing 99% with more than 94 isolates or strains of PEDV from US and Korea. The sequence of the PEDV USA/Colorado/2013 isolate (accession number: KF272920) was selected to compare the sequence PEDV/Cotopaxi/2014 since it showed the highest score from the BLASTn analysis. Thus, the sequence PEDV/Cotopaxi/2014 showed four nucleotide changes 1093AxG, 2454CxT, 3051CxT, and 3607CxT when both sequences were compared. Only two of these mutations led to amino acid changes when the deduced sequence was analyzed. The sequence PEDV/Cotopaxi/2014 showed the changes 360SxG and 1203LxF compared with the sequence of PEDV isolate USA/Colorado/2013. These mutations were not located at the neutralizing epitopes (SS2, SS6, or 2C10) [37]. Therefore, associations with possible adaptive advantages caused by escaping to the immune response of the host cannot be suggested. The mutation 360SxG was located into the N-terminal domain (NTD) of S1 (Figure 1). Even though this domain has not been directly linked to PEDV tropism and functionality as in transmissible gastroenteritis virus and murine hepatitis virus [38], it is recognized that it can influence virus infectivity [30]. Therefore, a mutation of serine in this domain could lead to an increase of the infectivity of the viral strains with this mutation, since serine is recognized as a catalytic residue of the trypsin (enzyme involved in the entry of the virus into the host cell in the digestive tract). Nevertheless, further virulence studies will be required to verify this role.

**Figure 2:**
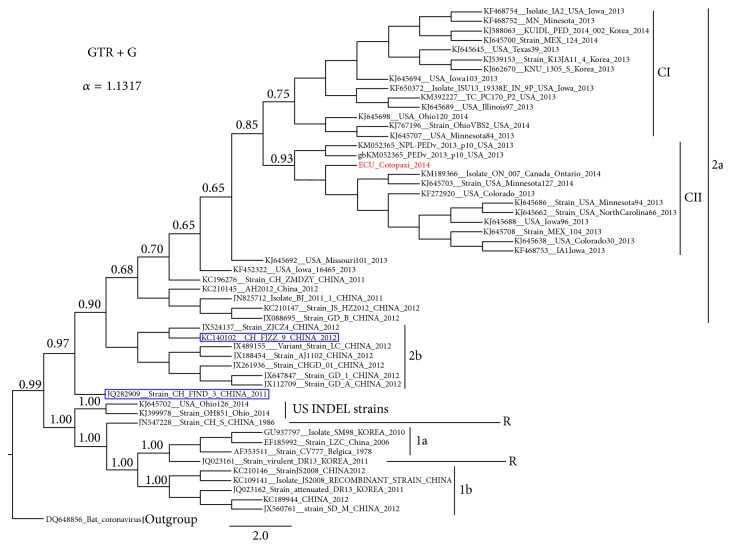
*Phylogenetic tree of PEDV based on complete S gene.* The best-fit model and the shape parameter of the gamma distribution (alpha) for the tree are indicated in the upper-left side. The numbers at a node are posterior probability values estimated. All different genogroups are denoted; the different clades CI and CII previously described by Vlasova et al. [28] into the genogroup 2a are also denoted. The sequence of PEDV/Cotopaxi/2014 is highlighted in red. Blue rectangles denote PEDV strains previously classified as 2a in Zhang et al. [31] (see Figure  1B in Zhang et al. [31]; the strains were clearly grouped into genogroup 2b but were denoted as 2a). The US INDEL sequences were also denoted.

**Figure 3:**
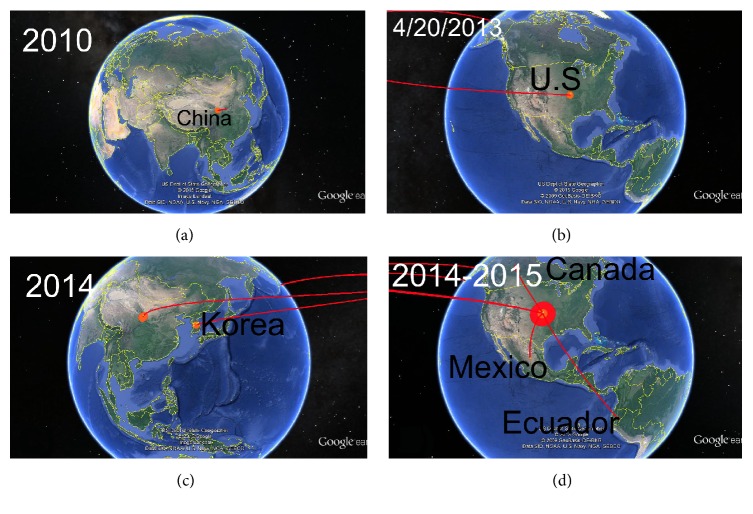
*Temporal dynamics of spatial PEDV diffusion.* Temporal distribution of PEDV; only rates supported by a BF of >5 were considered significant. The map was directly taken from the output file of the spread software and visualized using Google Earth (whole video included as Video S1). The emergence of the PDEV strains in China is represented in panel (a), the arrival to US in 2013 is shown in panel (b), and the posterior distribution of the PEDV strains-like US is represented in panels (c) and (d).

The phylogenetic relationships among the PEDV strains were reconstructed based on complete S gene by means of NJ, ML, and BI analyses. All algorithms yielded congruent results showing the same topologies, which was supported by moderate to high confidence values given by the bootstrap percentage and the posterior probability (Supplementary Material Figure S1). Even though the tree yielded by BI was the best, the statistical support for this tree was not significantly different from the NJ or ML trees (Supplementary Material Table S2). Thus, all topologies obtained showed two highly divergent genogroups (2a and 2b) (Supplementary Material Figure S1). For a better visualization of the results, a short tree obtained from 60 PEDV strains, representative of all genogroups or clades, was built by BI (Figure 2). Thus, the short tree showed the same distribution than the full tree (Supplementary Material Figure S1 and Figure 2). In Figure 2, two clades belonging to the PEDV genogroups 2a and 2b can be clearly observed. In addition, from the genogroup 2a, two main clades (I and II) previously described by Vlasova et al. [28] were also obtained (Figure 2). The sequence from the PED-outbreak in Ecuador PEDV/Cotopaxi/2014 was grouped into the clade II of PEDV genogroup 2a together with other sequences of isolates from Mexico, Canada, and United States (Figure 2). This group was highly supported by 0.93 posterior probability value (Figure 2). After the PED US outbreak, the virus rapidly spread to Canada and Mexico [4]. Whilst contaminated food by spray-dried porcine plasma positive to PEDV genome is pointed out as a possible way for introducing PEDV from US to Canada [18], the legal movement of pigs is suggested as the source of introduction to Mexico (http://www.thepigsite.com/swinenews/36693/mexico-reports-83-outbreaks-of-pedv-to-oie/). In the group of sequences in which PEDV/Cotopaxi/2014 was included, the nearest country to Ecuador is Mexico, indicating that the movement (legal or illegal) of animals between Mexico and Ecuador could be a possible source of introduction of the virus to Ecuador. However, evidence about this possible link has not been found. Phylogeographic reconstruction identified a specific location for the root of the tree with posterior probabilities for state sp = 0.81 for the locality of China (Supplementary Material Figure S2). The phylogeographic study revealed the emergence of the Chinese PEDV strains followed by spreading to US in 2013 (Figures 3(a) and 3(b), Supplementary Material Video S1). Huang et al. [17] previously observed this result describing the US PED-outbreak as an intercontinental transmission of the Chinese strains. After introducing the Chinese PEDV strains to US the virus spread from US to Korea (Figure 3(c), Supplementary Material Video S1). S. Lee and C. Lee [39] reported the circulation of new PEDV strains in South Korea that were genetically like PEDV strains that affected United States during 2013. This result suggested that the recent strains from South Korea might have been originated in the United States, caused by the importation of pig breeding stock during or after the sudden emergence of PEDV in the United States [39]. During 2010-2011, more than one-third of the total pig population in South Korea was slaughtered as control measure to foot-and-mouth disease outbreaks; thus a huge importation of breeding pigs from US was carried out without a proper implementation of a vaccination policy [39]. The phylogeographic study also revealed the introduction of the virus in Canada, Mexico, and Ecuador directly from the US (Figure 3(d) and Supplementary Material Video S1). This result frames the most probable route of entry of PEDV to Ecuador from US. The sources of imports of live swine in Ecuador in 2014 were mainly from Chile and US (http://data.trendeconomy.com/trade/Ecuador/Import?commodity=0103). Thus, this movement of pigs is suggested as the principal way for introducing PEDV to Ecuador.

Because this is the first introduction of PEDV in Ecuador swine herds, a fast spread of the virus throughout the country is expected, especially because a vaccination policy against PEDV has not been implemented yet. Therefore, additional studies to decipher how the virus will be disseminated in Ecuador and to other South American countries will be required.

## 4. Conclusion

In this study, a rigorous measurement of the global phylogeographic approach for PEDV strains was performed based on complete S gene sequences. The present work is the first study providing evidences that PEDV strains are circulating into Ecuador swine herds and revealing the molecular characteristic of the PEDV isolate in the South American region. The spatial analyses suggested that these strains were introduced to Ecuador by an importation from US.

## Figures and Tables

**Table 1:** Primers used for amplification of whole S gene and sequencing of PEDV/Cotopaxi/2014.

Primer	Sequence 5′-3′	Nucleotide position^*∗*^	Tm°C	Purpose	Product size (bp)
S5F	TCTTCTGGCGTAATTCCACA	20408–20427	56.86	Amplification (fragment 1)	1216
S1220R	TGAGCCTTCAGCAAGAATGA	21623–21604	57.13		
S621F	GGCCCCACTGCTAATAATGA	21024–21043	57.34	Amplification (fragment 2)	1399
S2019R	TGGTACAGGCACTAGCCAAA	22422–22403	58.94		
S1441F	TCAATGGGTTTGGATACTTGC	21844–21864	56.49	Amplification (fragment 3)	1391
S2831R	TCCATCACCATTAAACGAACT	23234–23214	55.22		
S2219 F	TTCTAGCTTTTTGGCAGGTG	22622–22641	56.24	Amplification (fragment 4)	1409
S3627R	CATCACCACCACAAAAACCA	24030–24011	56.73		
DPS3045 F	GGTGTTGTTGACGCTGAGAA	23448–23467	58.7	Amplification (fragment 5)	1377
DP4421R	TGACAACTGTGTCAATCGTGT	24824–24804	58.1		
426R	GGCCAGCACAGTACCAAGTT	20829–20810	58.7	Sequencing	-
1220R	TGAGCCTTCAGCAAGAATGA	21623–21604	60.54	Sequencing	-
3045F	GGTGTTGTTGACGCTGAGAA	23448–23467	57.13	Sequencing	-

^*∗*^The position of nucleotide corresponding to PED strain GenBank accession number KM392227.

## Supplementary Materials

Supplementary Materials Table S1. PEDV sequences of the complete S gene used in the current study (xls). Table S2. Comparison of topologies obtained for the complete S gene using ML, NJ, and BI methods. Figure S1. Phylogenetic tree obtained from the complete S gene and all sequences collected (Table S1) (TIFF). Figure S2. Maximum credibility phylogeographic tree. The posterior probabilities for state were denoted (TIFF). Video S1. Dynamics of spatial PEDV diffusion. The most probable temporal distribution of PEDV is provided; only rates supported by a BF of >5 were considered significant.
