# Supplementary material for: Tracking the Origin and Deciphering the Phylogenetic Relationship of Porcine Epidemic Diarrhea Virus in Ecuador
Source: Biomed Res Int. 2017 Dec 12;2017:2978718. doi: 10.1155/2017/2978718 (PMC5742880; doi:10.1155/2017/2978718)
Supplement: Supplementary Materials — Table S1. PEDV sequences of the complete S gene used in the current study (xls). Table S2. Comparison of topologies obtained for the complete S gene using ML, NJ, and BI methods. Figure S1. Phylogenetic tree obtained from the complete S gene and all sequences collected (Table S1) (TIFF). Figure S2. Maximum credibility phylogeographic tree. The posterior probabilities for state were denoted (TIFF). Video S1. Dynamics of spatial PEDV diffusion. The most probable temporal distribution of PEDV is provided; only rates supported by a BF of >5 were considered significant. [file 2978718.f1.zip › 2978718.f1/2978718.f1.pdf]

Tracking the origin and deciphering the phylogenetic relationships of porcine epidemic diarrhea virus in Ecuador.

Maritza Barrera[a,b], Ana Garrido-Haro[a], María S. Vaca[a], Danilo Granda[a], Lester J. Pérez[c*]

[a]*Laboratorio de Biología Molecular. Agencia Ecuatoriana de Aseguramiento de Calidad del Agro (Agrocalidad), Av. Interoceánica Km. 14.5, La Granja MAGAP, Tumbaco, Pichincha, Ecuador.*

[b]*Proyecto Prometeo. Secretaria Nacional de Educación Superior, Ciencia, Tecnología e Innovación-SENESCYT, Casa Patrimonial, calle 9 de Octubre y Ramírez Dávalos, Quito, Pichincha.*

[c] *Dalhousie Medicine New Brunswick (DMNB), Saint John, New Brunswick, E2L4L5, Canada.*

*Corresponding Author: +1 506-636-6977, Fax:+1 506-636-6258.

E-mail address: lester.perez@dal.ca (Pérez, L.J.)

Supplementary material

Table S1. Sequence used for phylogenetic inferences and phylogeographic approaches

GenBank Assession No.	Year of collection	Country	Isolate/strain
AB548618		Japan	83P-5
AB548619		Japan	83P-5 34th p
AB548620		Japan	83P-5 61 th p
AB548621		Japan	83P-5 6100th p
AB548622		Japan	KH
AB548623		Japan	NK
AB548624		Japan	MK
AB857233	2012	China	GDS01
AB857234	2013	China	GDS02
AB857235	2013	China	GDS03
AY653204		China	JS-2004-2
DQ462404		South Korea	DR13(atenuada)gen S
DQ862099		South Korea	DR13(atenuada)Genoma
JN315706	2011	China	CH/FJND-2/2011
JN543367	2011	China	CH/FJND-1/2011
JN599150	1994	China	CV777
JN825706	2011	China	BJ-2011-2
JN825707	2011	China	HB-2011-1
JN825708	2011	China	HB-2011-2
JN825709	2011	China	HB-2011-3
JN825710	2011	China	ZJ-2011-1
JN825711	2011	China	ZJ-2011-2
JN825712*	2011	China	BJ-2011-1
JN980698	2011	China	CHGD-01
JQ239429	2011	China	CH1
JQ239430	2011	China	CH2
JQ239431	2011	China	CH3
JQ239432	2011	China	CH4
JQ239433	2011	China	CH5
JQ239434	2011	China	CH6
JQ239435	2011	China	CH7
JQ239436	2011	China	CH8
JN381492	2011	China	CH/FJND-3/2011

JQ517274	2011	China	HuN
JQ627653	2011	China	CH/AY/11
JQ627654	2011	China	CH/CG/11
JQ638915	2011	China	CH/GD/2011
JQ638916	2011	China	CH/HLJHH/2011
JQ638917	2011	China	CH/SDLY/2011
JQ638918	2011	China	CH/HBBD/2011
JQ638919	2011	China	CH/SDQD/2011
JQ638920	2011	China	CH/JLCC/2011
JQ638921	2011	China	CH/BJSY/2011
JQ638922	2011	China	CH/HBQHD/2011
JQ638923	2011	China	CH/JLGZL/2011
JQ638924	2011	China	CH/JL/2011
JQ979287	2011	China	CH9-FJ
JQ979288	2011	China	CH13-GX
JQ979289	2011	China	CH17-GZ
JQ979290	2011	China	CH22-JS
JQ979291	2011	China	CH18-Hainan
JX018179	2012	China	CH/GXNN/2012
JX018180	2012	China	CH/YNKM/2012
JX018181	2012	China	CH/AHHF/2012
JX018182	2012	China	CH/AHHF-2/2012
JX018183	2012	China	CH/HBSN/2012
JX070671	2012	China	CH/FJXM-1/2012
JX070672	2012	China	CH/FJXM-2/2012
JX112709	2012	China	GD-A
JX163294	2012	China	HBMC2012
JX188454	2011	China	AJ1102
JX258672	2012	China	FQ/FJ/2012
JX261936	2011	China	CHGD-01
JX435298	2011	China	BJ-2011-3
JX435299	2012	China	BJ-2012-1
JX435300	2012	China	BJ-2012-2
JX435301	2011	China	HB-2011-4
JX435302	2012	China	HB-2012-1
JX435303	2012	China	HB-2012-2

JX435304	2012	China	HB-2012-3
JX435305	2012	China	HB-2012-4
JX489155	2011	China	LC
JX524137	2011	China	ZJCZ4
JX647847	2011	China	GD-1
KC196276*	2011	China	CH/ZMDZY/11
KC210147*	2012	China	JS-HZ2012
KC242898	2012	China	HeN12-20122
KC242899	2012	China	HB1-20122
KC242900	2012	China	HB2-20122
KC242902	2012	China	HB3-20123
KC242903	2012	China	ZJ17-20125
KC242904	2012	China	YN2-20127
KC242905	2012	China	FJ5-20128
KC242906	2012	China	FJZZ9-20128
KC242907	2012	China	JX4-20129
KC242908	2012	China	HN4-20129
KC242897	2011	China	FJZZ1-2011
KC242901	2012	China	AH2-20123
KC242911	2012	China	HN6-201211
KC242912	2012	China	HN8-201211
KC879275	2011	South Korea	AS01
KC879276	2012	South Korea	AS02
KC879277	2012	South Korea	AS03
KC879278	2011	South Korea	NJ01
KC879279	2012	South Korea	NJ02
KC879280	2011	South Korea	AD01
KC879281	2012	South Korea	AD02
KC879282	2012	South Korea	AD03
KF177254	2012	China	JY5C
KF177257	2012	China	YJ3F
KF177258	2012	China	YJ7C
KF272920*	2013	USA	USA/Colorado/2013
KF294254	2013	China	HZXS-1
KF294255	2013	China	HZXS-2
KF294256	2012	China	GJL

KF384500	2012	China	CH/GDGZ/2012
KF452322*	2013	USA	USA/Iowa/16465/2013
KF468752*	2013	USA	MN
KF468753*	2013	USA	IA1
KF468754*	2013	USA	IA1
KF468755	2013	China	XS2013
KF546800	2012	China	GDFS/2011
KF546801	2012	China	GDQY/2011
KF546802	2012	China	GDDG/2011
KF546803	2012	China	GDJM/2012
KF546804	2012	China	GDZQ/2012
KF577730	2013	China	YNKM/2012
KF577731	2013	China	GDMZ/2013
KF577732	2013	China	GDGZ/2013
KF601195	2013	China	GXLZ/2013
KF601196	2013	China	JXGZ/2013
KF601197	2013	China	HNCZ/2013
KF601198	2013	China	JXJA/2013
KF601199	2013	China	GXHZ/2013
KF601200	2013	China	GDEP/2013
KF601201	2013	China	GXNN/2013
KF650372*	2013	USA	ISU13-19338E-IN-passage9
KF650373	2013	USA	ISU13-22038-Iahomogenate
KF650374	2013	USA	ISU13-22038-IA-passage3,
KF650375	2013	USA	ISU13-22038-IA-passage9
KF738258	2012	China	GD120330
KF738259	2013	China	JS130604
KF738260	2013	China	JX130506
KF738261	2013	China	SD130502
KF738262	2013	China	SD130611
KF738263	2012	China	SH121229
KF738264	2012	China	ZJ121030
KF760557	2013	China	CH/JX-1/2013
KF761675	2013	China	CH/YNKM-8/2013
KF779469	2013	South Korea	MF3809/2008/South Korea
KF840537	2012	China	CH/ZJCX-1/2012
KF840538	2012	China	CH/ZJHY-2/2012

KF840539	2012	China	CH/ZJJS-Z/2012
KF840540	2012	China	CH/ZJJS-1Z/2012
KF840541	2012	China	CH/JXZS-3H/2012
KF840542	2012	China	CH/JXZS-2H/2012
KF840543	2012	China	CH/ZJXS212/2012
KF840544	2013	China	CH/ZJHZHY-6/2013
KF840545	2012	China	CH/JXJDZ-F/2012
KF840546	2012	China	CH/ZJJS-2Z/2012
KF840547	2012	China	CH/JXZS-3L
KF738258	2013	China	GD120330
KF738259	2013	China	JS130604
KF738260	2013	China	JX130506
KF738261	2013	China	SD130502
KF738262	2013	China	SD130611
KF738263	2012	China	SH121229
KF738264	2012	China	ZJ121030
KF760557	2013	China	CH/JX-1/2013
KF761675	2013	China	CH/YNKM-8/2013
KF779469	2008	South Korea	MF3809/2008/South Korea
KF840537	2012	China	CH/ZJCX-1/2012
KF840538	2012	China	CH/ZJHY-2/2012
KF840539	2012	China	CH/ZJJS-Z/2012
KF840540	2012	China	CH/ZJJS-1Z/2012
KF840541	2012	China	CH/JXZS-3H/2012
KF840542	2012	China	CH/JXZS-2H/2012
KF840543	2012	China	CH/ZJXS212/2012
KF840544	2013	China	CH/ZJHZHY-6/2013
KF840545	2012	China	CH/JXJDZ-F/2012
KF840546	2012	China	CH/ZJJS-2Z/2012
KF840547	2012	China	CH/JXZS-3L
KF840548	2012	China	CH/JXZS-1223L
KF840549	2012	China	CH/SDZD-1/2012
KF840550	2012	China	CH/SDZD-2/2012
KF840551	2012	China	CH/HuBWHYQ/2012
KF840552	2012	China	CH/ZJQZ-2/2012
KF840553.1_	2012	China	CH/ZJHZ-1C/2012
KF840555	2012	China	CH/JXJDZ-1/2012

KF840556	2012	China	CH/ZJJS-4X/2012
KF840561	2013	China	CH/JSZL-S2/2013
KF840562	2013	China	CH/JSZL-N2/2013
KF898124	1997	South Korea	KPEDV-9
KJ020932	2013	China	CHYJ130330
KJ184549	2013	USA	USA/KS/2013
KJ196348	2013	China	SHQP/YM/2013
KJ399978	2014	USA	OH851
KJ408801	2014	USA	OH1414
KJ526096	2013	China	CH/JX-2/2013
KJ539151	2013	South Korea	K13JA12-1
KJ539152	2013	South Korea	K13JA12-3
KJ539153	2013	South Korea	K13JA11-4
KJ584361	2013	USA	OH15962
KF452323.1_	2013	USA	USA/Indiana/17846/2013
KJ539154.1_	2014	South Korea	K14JB01
KJ646578	2013	China	FJ-ZP 2013
KJ646579	2013	China	FJ-CL 2013
KJ646580	2014	China	FJ-FQ 2014
KJ646581	2012	China	FJ-FQ1 2012
KJ646582	2012	China	FJ-FQ2 2012
KJ646583	2012	China	FJ-LY 2012
KJ646584	2013	China	FJ-LY 2013
KJ646585	2012	China	FJ-ND 2012
KJ646586	2013	China	FJ-ND 2013
KJ646587	2013	China	FJ-PT 2013
KJ646588	2013	China	FJ-QK 2013
KJ646589	2013	China	FJ-QZ 2013
KJ646590	2013	China	FJ-YX 2013
KJ646591	2014	China	FJ-ZP 2014
KJ662670	2013	South Korea	KNU-1305
KJ777677	2012	China	FGE_20140427
KJ777678	2012	China	CPGEN_20140427
KJ778616	2013	USA	NPL-PEDv/2013/P10
KM052365*	2013	USA	NPL-PEDv/2013/p10.1
KM108348	2014	South Korea	KPV1401
KM108349	2014	South Korea	KPV1402

KM108350	2014	South Korea	KPV1403
KM108351	2014	South Korea	KPV1404
KM108352	2014	South Korea	KPV1405
KM108353	2014	South Korea	KPV1406
KJ778615.1	2013	USA	NPL-PEDv/2013
KM189367.2	2014	Canada	ON-018
KM189368	2014	Canada	PEI-023
KM196109	2014	Canada	SK-030
KM196110	2014	Canada	2014-022; Bioassay Piglet # 30 3dpi
KM196111	2014	Canada	MB-021
KM213245		China	ZJ13LY1201
KM242131	2014	China	CH/GDZQ/2014
KM403155	2014	South Korea	KNU-1406-1
KM406178		China	LS1401
KM406179	2014	China	MY1402
KM406180	2014	China	DY1403
KM406181	2014	China	SH1404
KM406182	2014	China	MX1405
KM406183	2014	China	PZ1406
KM406184	2014	China	PJ1407
KM609203	2012	China	PEDV-1C
KM609204	2011	China	PEDV-7C
KM609205		China	PEDV-8C
KM609206	2012	China	PEDV-10F
KM609207	2011	China	PEDV-14
KM609208	2012	China	PEDV-15F
KM609209	2013	China	PEDV-CHZ
KM609210	2014	China	PEDV-LY
KM609211	2014	China	PEDV-LS
KM609212	2014	China	PEDV-LYG
KM609213	2014	China	PEDV-WS
KP276244.1	2014	Taiwan	TW-Chiayi-24
KP276247	2014	Taiwan	TW-Yunlin-82
KP276248	2014	Taiwan	TW-Yunlin-91
KP276249	2014	Taiwan	TW-Yunlin-71
KP276250	2014	Taiwan	TW-Pingtung-63
KP276251	2014	Taiwan	TW-Pingtung-41

KP276252	2014	Taiwan	TW-Pingtung-52
KP455313	2013	Viet Nam	HUA-PED45
KP455314	2013	Viet Nam	HUA-PED47
KP455315	2013	Viet Nam	HUA-PED55
KP455316	2014	Viet Nam	HUA-PED58
KP455317	2014	Viet Nam	HUA-PED60
KP455318	2014	Viet Nam	HUA-PED63
KP455319	2014	Viet Nam	HUA-PED67
KP455320	2014	Viet Nam	HUA-PED68
KR003452	2015	Belgium	15V010/BEL/2015
KR011756	2014	France	FR/001/2014
KR296663	2015	China	CH-GGC-01-2015
KR296664	2015	China	CH-GLC-02-2015
KR296665	2014	China	CH-GLC-12-2014
KR296666	2015	China	CH-HFEC-01-2015
KR296667	2015	China	CH-HGC-01-2015
KR296668	2015	China	CH-HSYKFC-01-2015
KR296669	2015	China	CH-JPYC-02-2015
KR296670	2015	China	CH-QTC-01-2015
KR296671	2015	China	CH-SBC-03-2015
KR296672	2014	China	CH-SFC-12-2014
KR296673	2014	China	CH-SHC-12-2014
KR296674	2014	China	CH-STC-12-2014
KR296675	2014	China	CH-TPC-12-2014
KR296676	2015	China	CH-WTC-02-2015
KR296677	2015	China	CH-XBC-01-2015
KR296678	2015	China	CH-YGC-01-2015
KR296679	2015	China	CH-ZSC-01-2015
KR296680	2015	China	CH-ZWBZa-01-2015
KR296681	2015	China	CH-ZWBZb-01-201
KR296682	2015	China	CH-ZWC-01-2015
KR296683	2015	China	CH-CLC-01-2015
KM213246		China	SH131202
EF185992		China	LZC
JN547228	1986	China	CH/S
JX560761	2012	China	SD-M
KF840557	2012	China	CH/ZJQZ-2w/2012

KF840558	2012	China	CH/ZJDX-1/2012
KJ158152	2011	China	AH-M
KM213244		China	ZJ14NB0401
KM213247		China	ZJ13SX1101
KM213248		China	ZJ13XS0401
KM213249		China	ZJ14HZ0301
JX088695*	2012	China	Strain-GD-B
KC210145*	2012	China	AH2012
KF468754*	2013	USA	Isolate-IA2
KM392227*	2013	USA	TC-PC170-P2
KM189366*	2014	Canada	Isolate-ON-007
KJ588063*	2014	Korea	KUIDL-PED 2014-002
KJ767196*	2014	USA	Strain-OhioVBS2
KJ645707*	2013	USA	USA-Minnesota84
KJ645703*	2014	USA	Strain-USA-Minnesota127
KJ645698*	2014	USA	USA-Ohio120
KJ645694*	2013	USA	USA-Iowa103
KJ645692*	2013	USA	USA-Missouri101
KJ645689*	2013	USA	USA-Illinois97
KJ645688*	2013	USA	USA-Iowa96
KJ645686*	2013	USA	Strain-USA-Minnesota94
KJ645662*	2013	USA	Strain-USA-NorthCarolina66
KJ645645*	2013	USA	USA-Texas39
KJ645638*	2013	USA	USA-Colorado30
KJ662670*	2013	South Korea	KNU-1305-S

Supplementary material

Table S2. Comparison of topologies obtained for the complete S gene using ML, NJ and BI methods.

Tree	Li	pKH	pSH	pRELL
S-BI	-12018.922	-1	-1	0.592
S-ML	-12021.478	0.129	0.719	0.078
S-NJ	-12032.416	0.285	0.540	0.270

Li: log-likelihoods
pKH: P value for KH normal test (Kishino & Hasegawa 1989).
pRELL: RELL bootstrap proportions (Kishino & Hasegawa 1989)
pSH: P value with multiple-comparison correction (MC in table 1 of Shimodaira & Hasegawa 1999)
(-1 for P values means N/A)

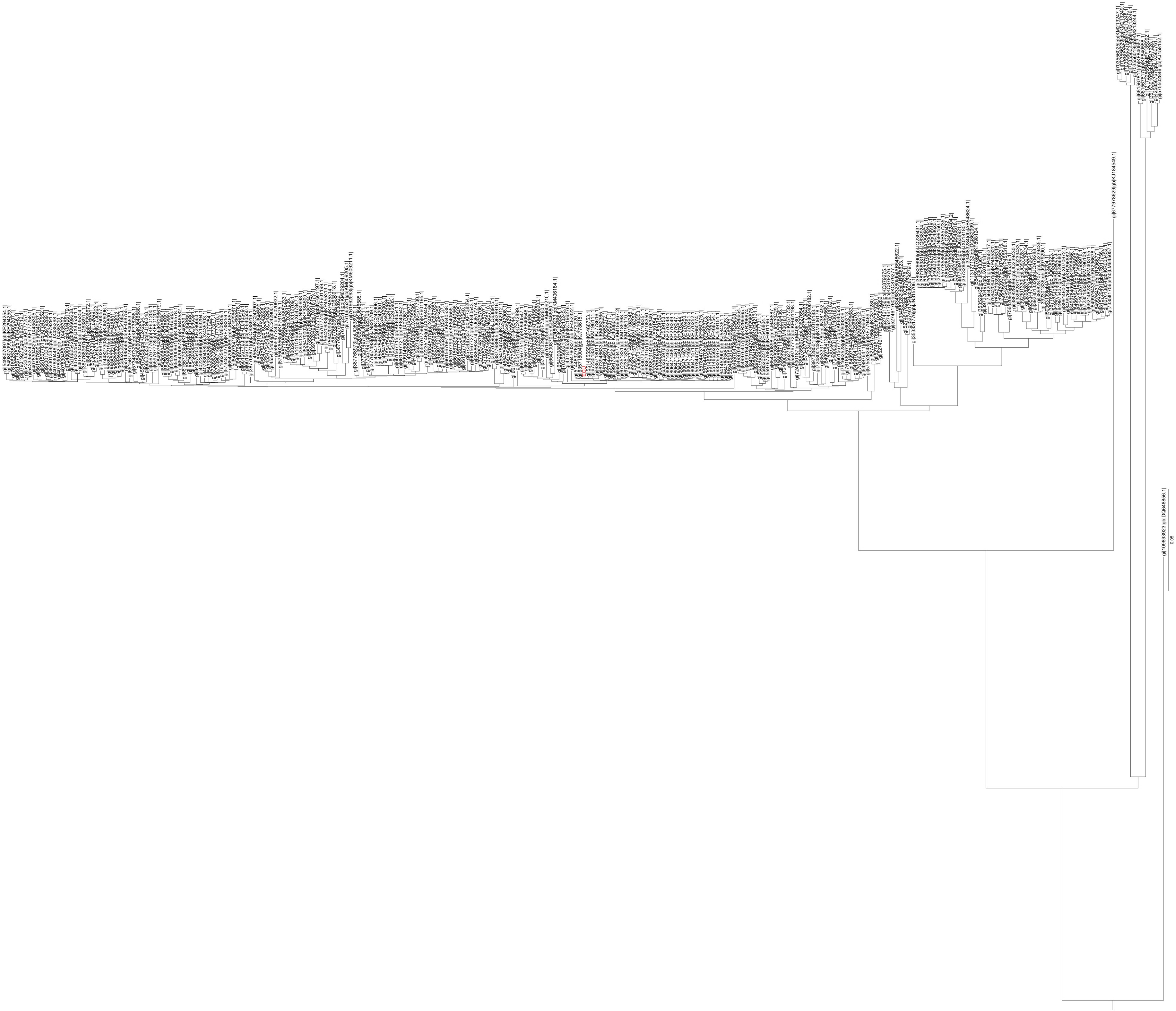
gi|677978629|gb|KJ184549.1|
gi|109893923|gb|DQ648856.1|
0.05

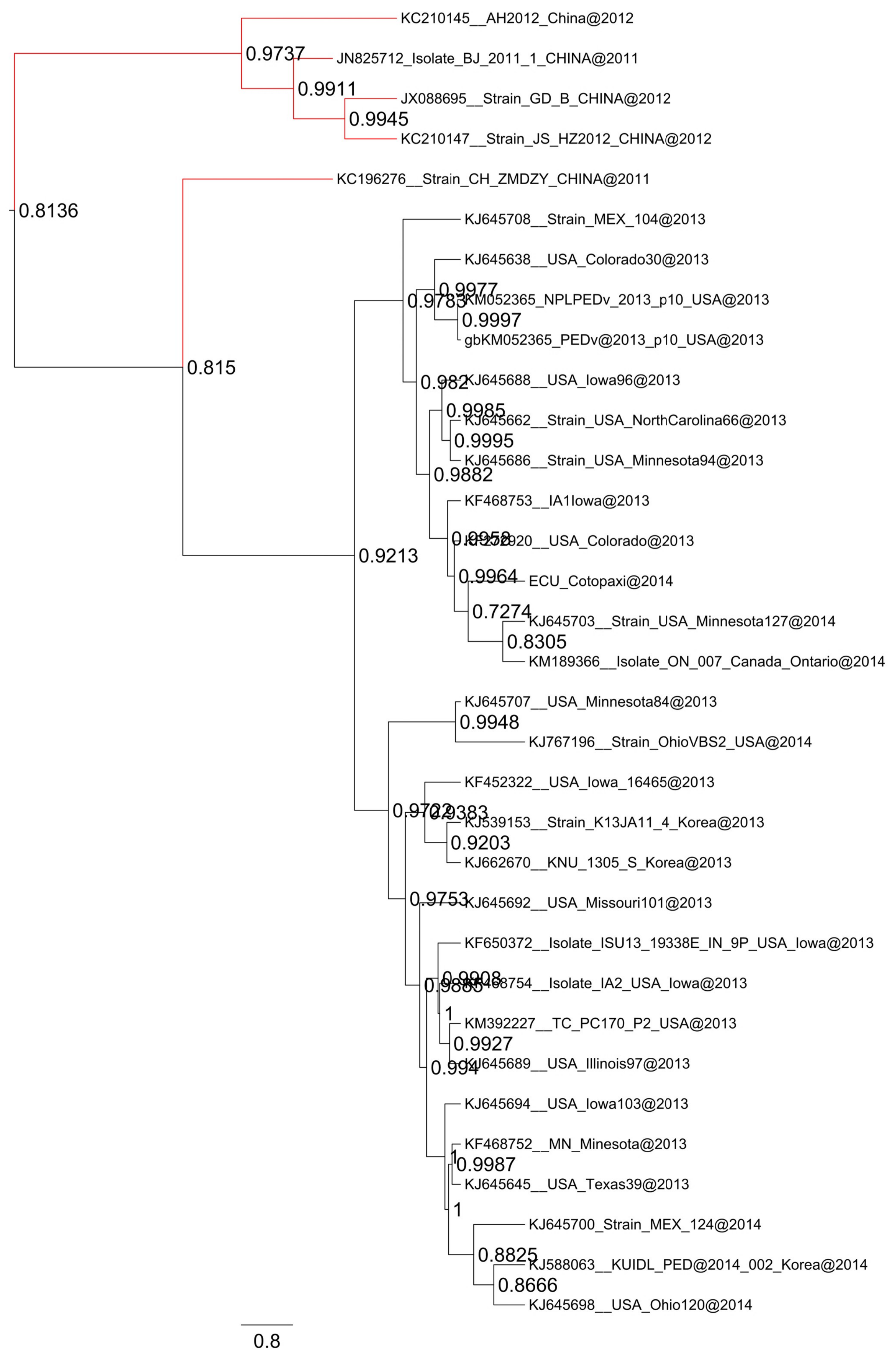

KC210145__AH2012_China@2012
JN825712_Isolate_BJ_2011_1_CHINA@2011
JX088695__Strain_GD_B_CHINA@2012
KC210147__Strain_JS_HZ2012_CHINA@2012
KC196276__Strain_CH_ZMDZY_CHINA@2011
KJ645708__Strain_MEX_104@2013
KJ645638__USA_Colorado30@2013
KM052365_NPLPEDv_2013_p10_USA@2013
gbKM052365_PEDv@2013_p10_USA@2013
KJ645688__USA_Iowa96@2013
KJ645662__Strain_USA_NorthCarolina66@2013
KJ645686__Strain_USA_Minnesota94@2013
KF468753__IA1Iowa@2013
USA_Colorado@2013
ECU_Cotopaxi@2014
KJ645703__Strain_USA_Minnesota127@2014
KM189366__Isolate_ON_007_Canada_Ontario@2014
KJ645707__USA_Minnesota84@2013
KJ767196__Strain_OhioVBS2_USA@2014
KF452322__USA_Iowa_16465@2013
KJ539153__Strain_K13JA11_4_Korea@2013
KJ662670__KNU_1305_S_Korea@2013
KJ645692__USA_Missouri101@2013
KF650372__Isolate_ISU13_19338E_IN_9P_USA_Iowa@2013
Isolate_IA2_USA_Iowa@2013
KM392227__TC_PC170_P2_USA@2013
KJ645689__USA_Illinois97@2013
KJ645694__USA_Iowa103@2013
KF468752__MN_Minesota@2013
KJ645645__USA_Texas39@2013
KJ645700_Strain_MEX_124@2014
KJ588063__KUIDL_PED@2014_002_Korea@2014
KJ645698__USA_Ohio120@2014
0.8136
0.9737
0.9911
0.9945
0.815
0.9213
0.9783
0.9977
0.9997
0.982
0.9985
0.9995
0.9882
0.9964
0.7274
0.8305
0.9948
0.9383
0.9203
0.9753
0.9908
0.9927
0.994
1
0.9987
0.8825
0.8666
0.8
